# Rank Awareness in Group-Sparse Recovery of Multi-Echo MR Images

**DOI:** 10.3390/s130303902

**Published:** 2013-03-20

**Authors:** Angshul Majumdar, Rabab Ward

**Affiliations:** 1 Indraprastha Institute of Information Technology, Delhi 110020, India; 2 Department of Electrical and Computer Engineering, University of British Columbia, Vancouver, BC V6T 1Z4, Canada; E-Mail: rababw@ece.ubc.ca

**Keywords:** MRI reconstruction, compressed sensing, low-rank matrix recovery

## Abstract

This work addresses the problem of recovering multi-echo T1 or T2 weighted images from their partial K-space scans. Recent studies have shown that the best results are obtained when all the multi-echo images are reconstructed by simultaneously exploiting their intra-image spatial redundancy and inter-echo correlation. The aforesaid studies either stack the vectorised images (formed by row or columns concatenation) as columns of a Multiple Measurement Vector (MMV) matrix or concatenate them as a long vector. Owing to the inter-image correlation, the thus formed MMV matrix or the long concatenated vector is row-sparse or group-sparse respectively in a transform domain (wavelets). Consequently the reconstruction problem was formulated as a row-sparse MMV recovery or a group-sparse vector recovery. In this work we show that when the multi-echo images are arranged in the MMV form, the thus formed matrix is low-rank. We show that better reconstruction accuracy can be obtained when the information about rank-deficiency is incorporated into the row/group sparse recovery problem. Mathematically, this leads to a constrained optimization problem where the objective function promotes the signal's groups-sparsity as well as its rank-deficiency; the objective function is minimized subject to data fidelity constraints. The experiments were carried out on *ex vivo* and *in vivo* T2 weighted images of a rat's spinal cord. Results show that this method yields considerably superior results than state-of-the-art reconstruction techniques.

## 1. Introduction

In multi-echo imaging, different images of the same cross-section are acquired by changing certain scan parameters, e.g., the echo times for T2 weighted images or the repetition times for T1 weighted images. The objective is to obtain images (of the same cross-section) with varying tissue contrasts. The details about the physics and techniques for acquiring these multi-echo MR images are found in [1]. In this work, we address the reconstruction of the images from their partial K-space samples.

Traditionally the K-space was obtained using full sampling on a uniform Cartesian grid. Each image was then reconstructed by applying the inverse Fast Fourier Transform (FFT). Full sampling of the K-space is however time consuming. Recent advances in Compressed Sensing (CS) allowed MRI researchers to reconstruct the MR images, almost perfectly, using partial, *i.e.*, not fully sampled, K-space scans [2,3]. Partial sampling of the K-space has the advantage of reducing the acquisition time. However, when the K-space is not fully sampled, the reconstruction problem becomes under-determined and prior information about the solution is needed for reconstructing the images.

Compressive Sampling (CS)-based MRI reconstruction has used the prior information that the images are spatially redundant, specifically that they have a sparse representation in a transform domain such as wavelets [2,3] or finite-differencing [2]. The techniques developed for single-echo MR images (such as [2,3]) are applied to each of the multi-echo images separately in order to reconstruct them from their partial K-space scans. However, this is not an optimal approach, and it was therefore argued in [4,5] that, since the multi-echo MR images are correlated, better reconstruction can be obtained when this correlation information is also exploited (along with the intra-image spatial redundancy). The reconstruction was formulated as a row-sparse Multiple Measurement Vector (MMV) recovery in [4] and as a group-sparsity vector recovery problem in [5]. The key difference between [4] and [5] is that, in [4] the same sampling mask was used in acquiring the K-space samples of every echo image while in [5] a different sampling mask was used for each echo.

This work is based on the concepts introduced in [4,5], mainly in order to achieve better reconstruction, both the inter-echo correlation and the intra-image spatial redundancy need to be exploited. In [4,5], the inter image correlation information assumed that the similarity amongst the images result in the locations of the images edges being the same over all the acquired echo images. Based on this assumption, the previous work showed that when the transform coefficients of the different echoes are stacked as columns of an MMV matrix or concatenated as a long vector, the matrix or vector thus formed is row-sparse or group-sparse respectively. Thus it required solving an optimization problem which promotes the signal's row/group sparsity.

Our work differs from its predecessors in the optimization problem used for reconstruction. As mentioned above, when the transform coefficients of the echo images are stacked in MMV form, the resulting matrix is row-sparse. Such a row-sparse matrix is low-rank as well (the rank is less than or equal to the number of non-zero rows). The key difference between this work and [4,5] is that it uses this extra information regarding rank deficiency of the MMV matrix along with row/group sparsity. Compared to [4,5] we use more information regarding the structure of the unknown signal (row/group sparsity and low-rank property) compared to [4,5] (only row/group sparsity).As mentioned above, owing to the partial sampling of the K-space, the reconstruction problem is under-determined and prior information regarding the solution is required. Intuitively, the greater the information we have regarding the unknown signal (solution), the better is the reconstruction. In the context of row-sparse MMV recovery, it has been theoretically proven in [6,7] that using the extra information that the MMV matrix has low rank (and not only the row-sparsity information), better reconstruction results can indeed be obtained (a row-sparse matrix will be obviously low-rank as well, and its rank will be less than or equal to the number of non-zero rows). Motivated by these studies, we propose to solve the multi-echo MRI reconstruction problem by formulating an optimization problem that exploits both the row/group sparsity and the low-rank properties of the unknown signals (to be reconstructed).

The problem of rank-deficient row-sparse MMV recovery has been studied before [6,7]. However multi-echo MRI reconstruction can only be formulated as a MMV recovery when the same sampling mask is used for collecting the K-space samples for all echoes. This is however a restrictive scenario. In general, we should be able to solve the problem even when different sampling masks are used for sampling the K-space data for every echo. This would require formulating the reconstruction as group-sparse vector recovery problem [5]. Thus for the general case (different sampling patterns for different echoes) we need to solve a rank-deficient group-sparse vector recovery problem. As this problem has not been encountered before there is no algorithm to solve it. This is another significant contribution of this paper. In the appendix, we derive an efficient solution to this rank-deficient group-sparse recovery problem.

The next section describes the theory behind the proposed method. Section 3 describes the experimental results. The conclusions of the work are discussed in Section 4. The derivation of the algorithm is relegated to the appendix since it may not be of interest to the majority of readers.

## 2. Theory

### 2.1. Literature Review

The previous work that is directly relevant to us is found in [4,5]. Multi-echo images pertain to the same cross section and only vary in tissue contrasts; the positions of edges (tissue boundaries) remain the same in all images. The wavelet transform encodes the discontinuities in images. The wavelet coefficients have high values along edges and are zeroes or near zeroes in smooth areas. Since the edges of the multi-echo images are aligned, the wavelet transform of the images will have high values at similar positions (along edges) in the different images. When the wavelet transform coefficients of different images are stacked as column vectors of a Multiple Measurement Vector (MMV) matrix, the resulting matrix is row-sparse (such a matrix is of size n × N, assuming that the wavelet coefficient vector is of length n and there are N such echo images). The MMV matrix is row-sparse since only those rows that correspond to edge positions have high values and the rest of the rows are zeroes or near zeroes [4]. Alternately the transform coefficients of the N echoes can be concatenated into a vector of length nN. The thus formed long vector can be grouped according to positions, *i.e.*, the j^th^ group is formed by collecting the j^th^ coefficient of the transform coefficients of each one of the N echoes. There will be n such groups of size N and the concatenated wavelet coefficient vector will be group-sparse [5]. Thus one can see that row-sparsity and group-sparsity are the same; these two forms arise out of the difference in arranging the vectors.

The aforesaid studies use two prior pieces of information—that the images are spatially redundant and are correlated with each other. The wavelet transform decorrelates the spatial redundancies in the individual images leading to a sparse representation; the fact that concatenated wavelet transform coefficients form a row-sparse matrix [4] or a group-sparse vector [5] arises from the inter-image correlation.

In this work, we follow the same assumptions as [4,5] but improve upon their results by incorporating more information regarding the solution. The K-space data acquisition model for the i^th^ echo image is as follows:

(1) $$y_{m\times 1}^{i}=F_{m\times 1}^{i}x_{m\times 1}^{i}+\eta_{m\times 1}^{i},m<n,i=1\ldots N$$

Here y is vector representing the acquired K-space data, $F_{m\times n}^{i}=R_{m\times n}^{i}F_{n\times n}$ is the Fourier mapping (corresponding to the sampling mask R^i^) of the image space into the K-space, x is the vector representing the image to be reconstructed, η is the system noise and N is the total number of multi-echo images. Since the K-space is partially sampled, solving *x* is an under-determined inverse problem.

For ease of expression, we will drop the matrix and vector dimensions in the rest of the text since they do not pose any ambiguity. There are two ways to arrange the multi-echo images which will result in slightly different reconstruction problems. In [4], it was assumed that the sampling mask remains the same for all the echoes (F^i^ = F for all i's), therefore the acquisition model could be expressed in the MMV form.

(2) Y=FX+N

Here Y is the matrix formed by vertically stacking the y^i^'s, X is the matrix formed by vertically stacking x^i^'s and N is formed by vertically stacking η^i^'s.

**Figure 1.**
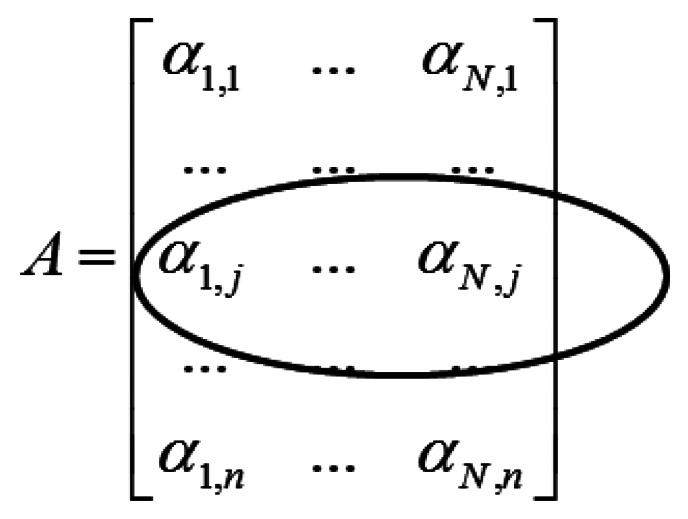
Row structure in MMV matrix.

Applying the wavelet transform (W) on X leads to a row-sparse MMV matrix A(=WX). This is because the resulting wavelet transform coefficients will only have high values along rows that correspond to edges in the multi-echo images. The MMV matrix is shown in Figure 1. The α_i_'s are the transform coefficient vectors.

The row-structure is highlighted in Figure 1. Only those rows that correspond to edges will have nonzero values; if the j^th^ row corresponds to an edge it will have non-zero values throughout the row. To recover the row-sparse MMV matrix A, the following optimization problem was proposed in [4]

(3) $$\underset{A}{\text{min}}{\left\|A\right\|}_{2,1}s.t.{\left\|Y-FW^{H}A\right\|}_{F}^{2}\leq ɛ$$

Here A = WX is the matrix formed by stacking the wavelet transform coefficients as columns, ‖.‖_2,1_ is a mixed norm defined as the sum of the *l_2_*-norms of the row-vectors and ‖.‖_F_ is the Frobenius norm (Euclidean norm of a matrix) and ε = mNσ^2^, where σ is the standard deviation of noise. The *l_2_*-norm along the row vector promotes a dense (not sparse) solution along the selected rows but the sum over the *l_2_*-norms promotes the selection of only a few rows [8].

The problem with this approach [4] is that the sampling mask should remain the same for all echoes. CS based recovery techniques demand maximum incoherence in sampling [9]. The method we discussed so far does not satisfy this requirement since all the echoes are sampled by the same mask. It has been shown in [5] that better reconstruction can be obtained if different sampling masks are used for the different echoes. This leads to a different arrangement of the wavelet coefficient vectors and leads to a group-sparsity promoting optimization [5].

In [5] the data acquisition model is represented concisely in the following form:

(4) y=Fx+η

where y is the vector formed by concatenating y^i^'s, F is the block diagonal matrix with F^i^'s in the diagonal blocks, x is the vector formed by concatenating x^i^'s, η is the vector formed by concatenating η^i^'s. Such a representation allows for different sampling masks for different echoes.

**Figure 2.**
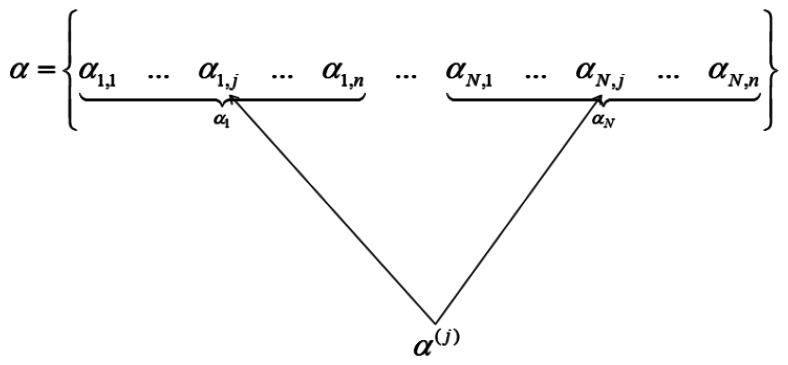
Grouping according to positions.

When the wavelet transform coefficients of all images are concatenated as vector α, then if all the coefficients corresponding to the same index in each image are grouped, only those groups that correspond to edges in the original image will have high valued coefficients, the rest will be zeroes or near about zeroes. This is shown in Figure 2.

We see how the transform coefficients are grouped according to original indices in Figure 2. If the position corresponds to an edge in the image, the j^th^ group (α^(j)^) will have high value. Thus solving the wavelet transform coefficients of the images turns out to be a group-sparse optimization problem. In [5] the following optimization problem is proposed for reconstruction of α:

(5) $$\underset{\alpha }{\text{min}}{\left\|\alpha \right\|}_{2,1}s.t.{\left\|y-\Phi \alpha \right\|}_{2}^{2}\leq ɛ$$

Here α is the vector formed by concatenating the wavelet transform coefficients of all the images, Φ is the block diagonal matrix consisting of F^i^W^H^ as diagonal elements and ε is as defined before in Equation (3). The mixed *l_2,1_*-norm is defined as the sum of *l_2_*-norms of the groups (grouped according to positions in this case). The idea behind the *l_2,1_*-norm is the same as before, the *l_2_*-norm on the groups promotes a dense(*i.e.*, non-sparse) solution within the groups but the sum over the *l_2_*-norms promotes selection of only a few groups [10].

The aim of this work is to reconstruct multi-echo MR images. But this is the intermediate step. In quantitative MRI, the final objective is to compute the T2 or T1 maps. Generally, these maps are computed by non-linear curve fitting of the multi-echo images. This work aims at computing these maps from partially sampled K-space measurements. In this work, we show that if we can faithfully recover the images by our proposed method, the computed maps can be computed fairly accurately as well. There is one work which skips the intermediate step of reconstructing the images and directly computes the maps from the under-sampled K-space measurements [11]. Here a learned dictionary is used to compute the maps directly. There is yet another work which computes the maps directly from the K-space samples by solving a non-linear inversion problem [12].

However the aforementioned techniques are not standard ones in quantitative MRI. In this work, we follow the more conventional approach. The multi-echo images are reconstructed from which the maps are computed via non-linear least squares fitting.

### 2.2. Proposed Method

Let us re look at the MMV model for multi-echo MR imaging [2]; we repeat the data acquisition model for the sake of convenience: *Y* = *FX* + *N*

Incorporating the wavelet transform into the data acquisition model we get:

(6) $$Y+FW^{H}A+N$$

In [4], the wavelet transform coefficients of the multi-echo images (A) were solved by solving a row-sparsity promoting optimization problem. However, it should be noticed that such a row-sparse matrix (A) is rank deficient as well—rank of matrix will be less than the number of non-zero rows. As mentioned above, the information regarding the rank deficiency was not incorporated into the reconstruction formulation in [4].

Since, the MMV matrix to be recovered is rank deficient; one can solve it via Nuclear-norm minimization [13]. The following problems need to be solved,

(7) $$\underset{A}{\text{min}}{\left\|A\right\|}_{NN}s.t.{\left\|Y-FW^{H}A\right\|}_{F}^{2}\leq ɛ$$

Here ‖.‖_NN_ represents the nuclear norm of the Matrix which is defined as the sum of singular values of the matrix. Solving the reconstruction of A by utilizing only its rank-deficient property (solving Nuclear-norm minimization) will not yield good results. This is because the fact that A is rank-deficient is an effect of A being primarily row-sparse; it is low rank since only a few rows are non-zeroes. Thus row-sparsity is a more fundamental property and without it the reconstruction will not be good. The best solution can be achieved when the information about both its row-sparsity and rank-deficiency is incorporated into the reconstruction formulation simultaneously. The straightforward way to fuse the two pieces of information (rank-deficiency and row-sparsity) is to solve the following optimization problem:

(8) $$\underset{A}{\text{min}}{\left\|A\right\|}_{2,1}+\gamma {\left\|A\right\|}_{NN}s.t.{\left\|Y-FW^{H}A\right\|}_{F}^{2}\leq ɛ$$

Here γ is the term controlling the relative importance of the row-sparsity and rank-deficiency. Intuitively Equation (8) is supposed to yield the best possible reconstruction result (compared to Equations (3) and (7)). This intuition is theoretically confirmed in [6,7]. They have shown that (assuming that the quality of reconstruction is kept constant), when both the row-sparse property and the low-rank property are exploited when recovering the signal then a lesser number of measurements (m) are required compared to the cases where only row-sparsity or rank-deficiency are exploited. In other words, when the same number of samples, is used, a better reconstruction quality is achievable if both row-sparsity and rank-deficiency are exploited. In [6], a greedy algorithm is proposed to solve Equation (8) while in [7] a non-convex optimization based formulation is proposed.

The problem with the MMV formulation (as discussed above) is that it does not allow for separate sampling masks for each echo. To accommodate separate masks, the data acquisition equation should be expressed as Equation (4). We repeat it for the sake of convenience: *y* = *Fx* + *η*.

When the Wavelet transform is incorporated into the data acquisition model, we have the following form:

(9) y=FΦα+η

In Equation (9), the wavelet coefficient vectors α_i_ = Wx^i^'s are concatenated to form α. A group-sparse optimization problem was proposed in [5] to solve said problem. However, we have seen in Equation (6), where the transform coefficients are stacked as columns of a matrix, the thus formed matrix is row-sparse. Both group-sparsity and row-sparsity arise from the fact that the multi-echo images are correlated with each other and have edges/discontinuities at similar positions. Therefore when the wavelet transform encodes these edges, there appear high valued coefficients only along the edges and zeroes (or near about zeroes) elsewhere (in smooth areas).

Group-sparsity and row-sparsity are two different ways of representing the same phenomenon. In this work, we use the extra information that the matrix formed by stacking the wavelet transform coefficients as its columns is a low-rank matrix. For row-sparse MMV recovery, the low-rank property is exploited as done in Equation (8). To use the low-rank property into the group-sparse formulation, we propose to formulate the reconstruction as the following problem:

(10) $$\underset{\alpha }{\text{min}}{\left\|\alpha \right\|}_{2,1}+\gamma {\left\|A\right\|}_{NN}s.t.{\left\|y-\Phi \alpha \right\|}_{2}^{2}\leq ɛ$$

Here the parameter γ also controls the relative importance of group-sparsity and rank-deficiency. The said optimization problem Equation (10) has not been encountered before in the literature; thus there is no algorithm to solve it. In this work, we derive an algorithm to solve this problem. As the mathematical details of the solution may not be of interest to many readers, the derivations has been delegated to the appendix.

Experts in medical physics pointed out that, for rigid tissues (like cartilages) portions of the tissues can have low relaxation times which will lead to change in the position of its edges across echoes. In such a situation, the transform coefficients will not have a common (sparse) support across echoes. As a result, the MMV matrix A Equation (6) or the concatenated vector α Equation (9) will not be exactly row-sparse or group-sparse, *i.e.*, there will be rows or groups that are not dense, there will be rows or groups that have zeroes within. In such a scenario our proposed method (as well as previous techniques [4,5]) may not yield good results at least in theory.

In such a case, the problem may be rectified by introducing non-convex row-sparsity or group-sparsity promoting norms:

(11) $$\underset{A}{\text{min}}{\left\|A\right\|}_{m,1}+\gamma {\left\|A\right\|}_{NN}s.t.{\left\|Y-FW^{H}A\right\|}_{F}^{2}\leq ɛ,0<m\leq 1$$

(12) $$\underset{\alpha }{\text{min}}{\left\|\alpha \right\|}_{m,1}+\gamma {\left\|A\right\|}_{NN}s.t.{\left\|Y-F\Phi \alpha \right\|}_{2}^{2}\leq ɛ,0<m\leq 1$$

Here, the non-convex norm ‖.‖*_1_* allows for sparse solutions within the rows or groups for *0* < *m* ≤ *1*. It is easy to incorporate solutions to such non-convex problems in the algorithm derived in the appendix. However, in this work, we only work with convex problems, and assume that the said scenario (changing positions of edges across echoes) does not arise. The main idea of this work is to show that if the assumptions used in [4,5] hold, how can we improve upon the reconstruction results by incorporating more information (low-rank property) into the reconstruction problem.

## 3. Results and Discussion

### Excised Spinal Cord

All animal experimental procedures were carried out in compliance with the guidelines of the Canadian Council for Animal Care and were approved by the institutional Animal Care Committee. One female Sprague-Dawley rat was obtained from a breeding facility at the University of British Columbia and acclimatized for seven days prior to the beginning of the study. Animal was deeply anaesthetized and perfused intracardially with phosphate buffered saline for 3 minutes followed by freshly hydrolysed paraformaldehyde (4%) in 0.1 M sodium phosphate buffer at pH 7.4. The 20 mm spinal cord centred at C5 level was then harvested and post-fixed in the same fixative. MRI experiments were carried out on a 7 T/30 cm bore animal MRI scanner (Bruker, Germany). Single slice multi-echo CPMG sequence [8] was used to acquire fully sampled k-space data from the excised spinal cord sample using a 5 turn, 13 mm inner diameter solenoid coil with 256 × 256 matrix size, TE/TR = 13.476/1,500 ms, 16 echoes, 2.56 cm field-of-view (FOV), 1 mm slice, and the excitation pulse phase *cycled* between 0° and 180°.

### *In Vivo* Imaging

A rectangular coil (22 × 19 mm) was surgically implanted over the lumbar spine (T13/L1) of a female Sprague-Dawley rat as described previously [9]. For MRI experiments, the animal was anaesthetized with isoflurane (5% induction, 2% maintenance) mixed with medical air and positioned supine in a specially designed holder. Respiratory rate and body temperature were monitored using an MRI compatible monitoring system (SA Instruments, Stony Brook, NY, USA). Heated circulating water was used to maintain the body temperature at 37 °C. Data was acquired using the same CPMG sequence but with slice thickness of 1.5 mm and in-plane resolution of 117 μm. The slice was positioned at T13/L1 level.

### Methodology

In this work we follow the same experimental methodology as in [4,5]. For both the *in vivo* and the *ex vivo* data, the groundtruth data consisted of the fully sampled K-space from which the images were reconstructed by inverse Fast Fourier Transform. We simulate variable density partial sampling of the K-space by randomly omitting lines in the frequency encoding direction. Three sampling patterns for 32, 48 and 64 lines in the phase-encode direction corresponds to sampling ratios 12.5%, 18.75% and 25% of the full K-space. For all the sampling patterns, a third of the total sampling lines are used to densely sample the center of the K-space. The rest of the sampling lines are spaced uniformly at random over the remaining K-space. Since most of the information in an MRI is concentrated in the low frequency region, such a sampling pattern ensures that this information is used in reconstruction. In [4], one sampling mask is generated and this is used to sample all the echoes. In [5], different sampling masks are generated for each echo. The difference between the sampling masks arises owing to the random positions of the 2/3rd sampling lines which are used to sample the K-space away from the center.

The algorithm used in this work requires certain parameters to be specified. In Equation (10), γ controls the relative importance of group-sparsity and rank-deficiency. This parameter has been fixed by the L-curve method [14]. This is a well known method to choose regularization parameters for MRI reconstruction problems [15]. By this method we found γ = 12.5 yields the best results.

We tested several families of wavelet transforms—Daubechies, fractional spline and complex dualtree. We found that the best results were obtained with the complex dualtree wavelets. Therefore all the results reported here use the complex dualtree wavelets as the sparsifying transform. For rank-deficient row-sparse MMV recovery we employed the optimization problem proposed in [7]. For rank-deficient group-sparse recovery we used the algorithm derived in this work.

### 3.1. Quantitative Results

We use the Signal-to-Noise ratio (SNR) as the metric for comparing the quantitative reconstruction results. Our first set of experiments constrains all the echoes to have the same sampling mask. This corresponds to the situation studied in [4]. In [4], row-sparse recovery was used for multi-echo MRI reconstruction. In this work, the results obtained using the proposed optimization problem Equation (8) is compared against those proposed in [4]. The results are shown in Tables 1 and 2 for *ex vivo* and *in vivo* data respectively.

The results experimentally validate the intuition behind this work—the more information we use for reconstruction, the better is the result. In [4], the information about row-sparsity was solely used; here we exploit the information that the signal to be recovered is rank-deficient as well. This leads to better reconstruction accuracy compared to the previous method.

In the next set of experiments, we use a different sampling mask for each echo. Here instead of using the MMV formulation, we use the proposed optimization problem Equation (10). Therefore we compare our work with [5]—which uses the group-sparsity promoting optimization problem Equation (5) to reconstruct the images.

Comparing Tables 1 and 2 to Tables 3 and 4, we notice that better results are obtained when different Fourier mappings are used for different echoes. Also we see that, better results are obtained by our proposed method since we use more information regarding the structure of the solution (group-sparsity and rank-deficiency compared to only group-sparsity as in [5]).

From Tables 1, 2, 3 and 4 what we observe is that for higher under-sampling ratios (lesser number of samples) the improvements in reconstruction accuracy from our proposed methods are more (around 1.9 dB for 32 lines), but the relative improvement in accuracy falls at higher sampling ratios (around 1.1 to 1.2 dB for 64 lines). This means that our proposal to incorporate information about rank-deficiency of the solution is useful for higher under-sampling only. This actually corroborates the theoretical findings in [6,7] where it has been proven that joint-sparse recovery is only going to yield better recovery results when the under-sampling ratio is high.

### 3.2. Qualitative Results

**Figure 3.**
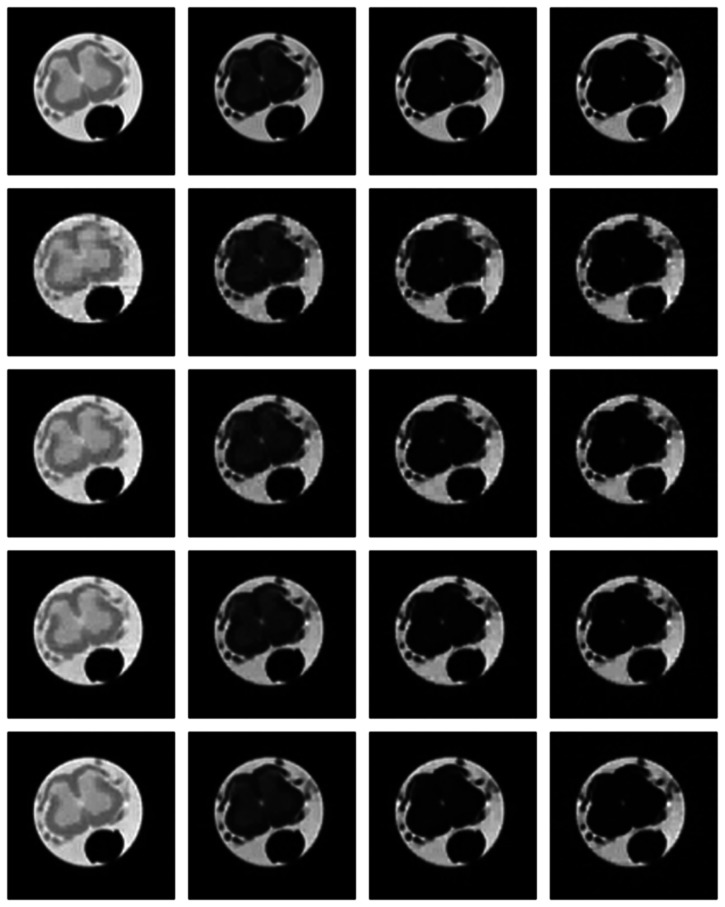
Images from *ex vivo* reconstruction. Top row—groundtruth; 2nd row—same sampling pattern, row-sparse recovery; 3rd row—same sampling pattern, rank-deficient row-sparse recovery; 4th row—different sampling pattern, group-sparse recovery; 5th row—different sampling pattern, rank-deficient group-sparse recovery.

**Figure 4.**
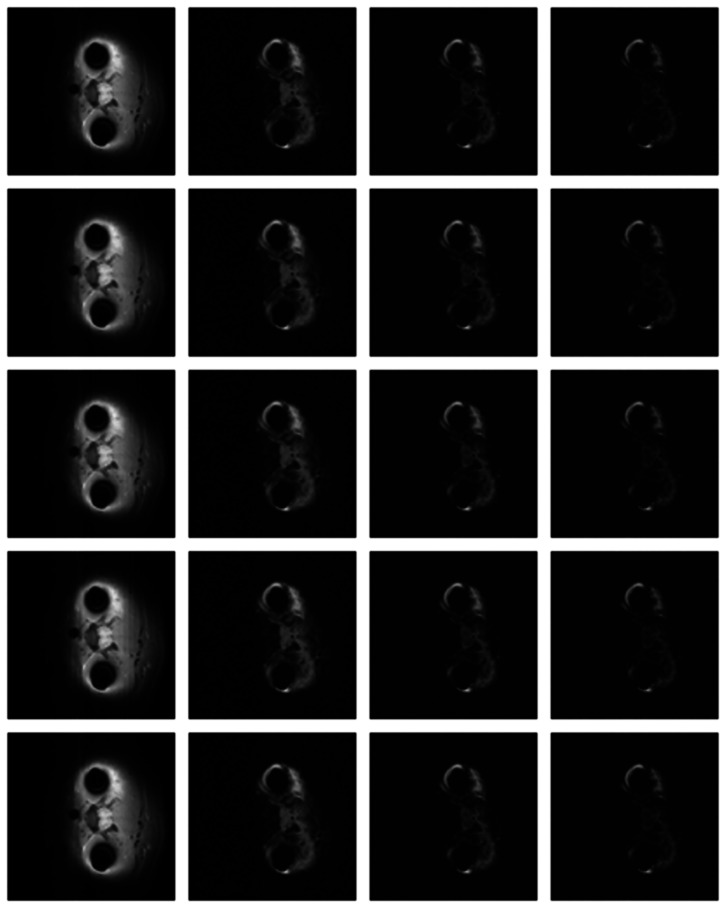
Images from *in vivo* reconstruction. Top row—groundtruth; 2nd row—same sampling pattern, row-sparse recovery; 3rd row—same sampling pattern, rank-deficient row-sparse recovery; 4th row—different sampling pattern, group-sparse recovery; 5th row—different sampling pattern, rank-deficient group-sparse recovery.

Numerical results do not always provide information about the qualitative nature of reconstruction. Thus, we provide reconstructed and difference images for visual inspection. Owing to limitations in space, we only show the results for 64 lines (this corresponds to a sampling ratio of 25%). Echo numbers 1, 5, 9 and 13 are shown in the following figures. In Figures 1 and 2, the *ex vivo* and *in vivo* reconstructed images are shown. In Figures 3 and 4, the difference images corresponding to *ex vivo* and *in vivo* imaging are shown respectively.

Looking closely at these figures, especially at the edges, one can see that the reconstruction quality progressively improves as one move from top to bottom. The worst results are obtained when the same Fourier map is used for all the echoes and simple row-sparse MMV recovery is used. The results improve slightly when rank-deficiency is introduced, but the improvement is only slight because the condition of incoherence is not satisfied when the same Fourier map is used for all the echoes. When different Fourier maps are used for the different echoes, the results from both group-sparse recovery and rank-deficient group-sparse recovery show improvement in the reconstruction quality; this is because of the better incoherence in the measurement operator. However, the best results are obtained when rank-deficient group-sparse recovery is used with different Fourier maps for different echoes.

The differences in the reconstruction quality are best evaluated from the difference images. The difference is formed by taking the absolute difference between the reconstructed and the groundtruth images. The contrast of the difference images is enhanced five times for visual clarity.

**Figure 5.**
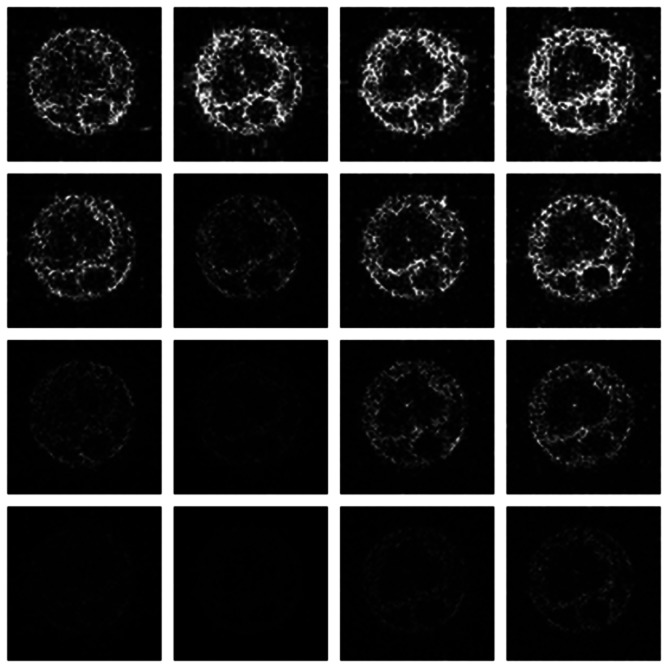
Difference Images from *ex vivo* reconstruction. Top row—same sampling pattern, row-sparse recovery; 2nd row—same sampling pattern, rank-deficient row-sparse recovery; 3rd row—different sampling pattern, group-sparse recovery; 4th row—different sampling pattern, rank-deficient group-sparse recovery.

**Figure 6.**
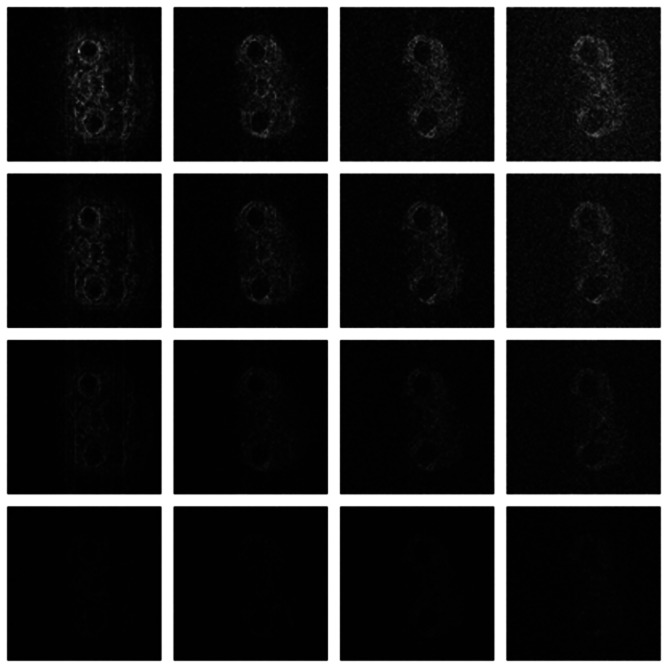
Difference Images from *In vivo* Reconstruction. Top row—same sampling pattern, row-sparse recovery; 2nd row—same sampling pattern, rank-deficient row-sparse recovery; 3rd row—different sampling pattern, group-sparse recovery; 4th row—different sampling pattern, rank-deficient group-sparse recovery.

The difference in the reconstruction quality is easily discernible in the difference images; see Figures 5 and 6. The difference images that are brighter indicate the presence of higher reconstruction error. We can see that group-sparse recovery using the same Fourier mapping on all the echoes yields the worst reconstruction while our proposed rank-deficient group-sparse recovery (different Fourier map for each echo) yields the best results.

This work assumes that if the multi-echo MR images reconstructed from partial K-space scans are similar to those from full K-space scans, the corresponding T2 maps will be similar as well. This assumption is validated experimentally. In the following figure, we show the T2 maps for the *ex vivo* and *in vivo* data. The CPMG data were processed with software procedures developed in-house using MATLAB R2009b. Regularized non-negative least square (NNLS) analysis [16] was used to calculate the continuous T2 distributions. For the *in vivo* data, only the portion corresponding to the spinal cord is fit.

**Figure 7.**
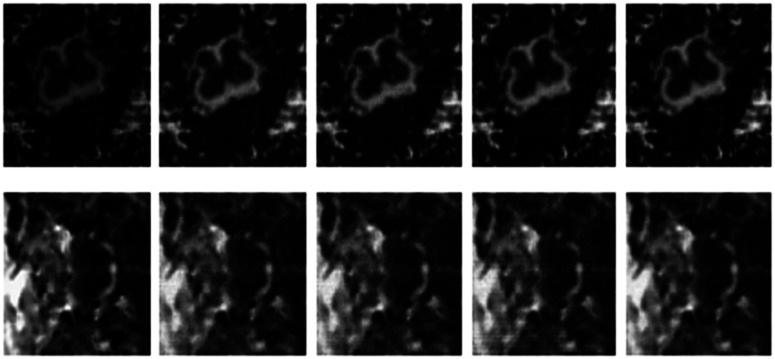
T2 maps for *Ex vivo* (top) and *In Vivo* (bottom) data; from left to right—Groundtruth, same sampling mask row-sparse recovery; same sampling mask, proposed rank-deficient row-sparse recovery; different sampling masks, group-sparse recovery; different sampling masks, proposed rank-deficient group-sparse recovery.

**Figure 8.**
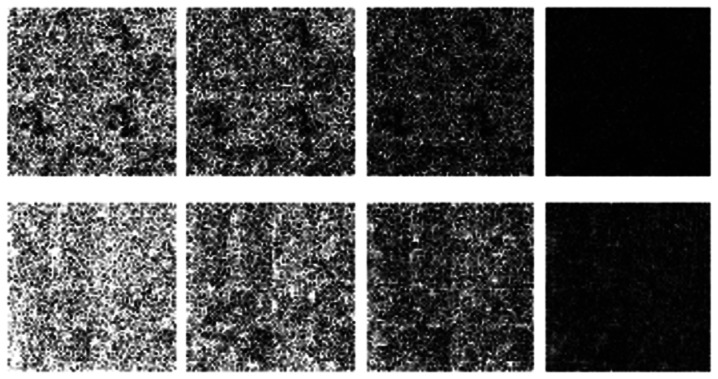
T2 difference maps for *Ex vivo* (top) and *In Vivo* (bottom) data; from left to right—same sampling mask row-sparse recovery; same sampling mask, proposed rank-deficient row-sparse recovery; different sampling masks, group-sparse recovery; different sampling masks, proposed rank-deficient group-sparse recovery.

From Figure 7, the maps reconstructed from partial K-space data look almost the same as the map reconstructed from full K-space sampling. The difference in quality is more clearly visible from the difference maps shown in Figure 8. The contrast of the difference images are enhanced 10 times for visual clarity. It can be seen that for the same masks (for all echoes) the difference image shows large reconstruction error (almost white image). The reconstruction result improves when different sampling masks are used. But in either case, our proposed method with mixed prior optimization yields better results than the corresponding former techniques [6,7]. The best results are obtained (almost no reconstruction error) when mixed prior optimization is used with different sampling masks for each echo. One can also notice that the in general the *ex vivo* difference images are darker (less reconstruction error) compared to the *in vivo* images. This owes to the fact that *in vivo* images are more difficult to reconstruct.

## 4. Conclusions

In this work we address the problem of multi-echo MRI reconstruction form under-sampled K-space data. The experiments are carried out on T2 weighted images but the methodology developed here is applicable to other modalities (such as T1 weighted imaging) as well. This builds on our earlier work [4,5], where we have argued that the best results for multi-echo imaging reconstruction are obtained by exploiting the inter-echo correlation along with the intra-image spatial redundancy. In the previous studies, the reconstruction problem was formulated either as a row-sparse Multiple Measurement Vector (MMV) recovery [4] (when the same Fourier mapping (sampling mask) was used for all echoes) or as a group-sparse vector recovery [5] (when different Fourier mappings are used for different echoes). Both [4,5] use Compressed Sensing (CS) based techniques which exploit the row/group sparsity of the transform domain coefficients in order to reconstruct the images. In this work, we propose an improvement. We identify that, when stacked as column vectors, the sparse transform coefficients of the multi-echo images constitute a matrix which is not only row/group sparse but is low-rank as well. The previous studies [4,5] only exploited the sparsity of transform coefficients for reconstruction. Here we proposed to use information about both sparsity (row/group) of the signal along with its rank-deficiency. As we use more information regarding the structure of the signal we need to reconstruct, we obtain better results.

In this work, we propose the reconstruction problem as a synthesis prior problem. In synthesis prior formulation, the sparse transform coefficients are solved. Previously we have found that better reconstruction can be obtained if the analysis prior problem is solved instead. In the analysis prior problem, the images are directly reconstructed (and not their transform coefficients) [5,15]. In the future, we wish to investigate whether the results can be further improved by using the analysis prior formulation instead of the synthesis prior formulation as proposed in this work.

The major limitation of this work (and the prior methods [4,5]) is in the imaging of rigid tissues like cartilages, where the relaxation times can be so short that the positions of the edges will shift in different echoes. This limits the applicability of our proposed method and further research needs to be carried out in order to tackle this problem.

## Figures and Tables

**Table 1.** *Ex vivo*: SNR for same Fourier mapping with all echoes.

**Recovery Method**	**32 Lines**	**48 Lines**	**64 Lines**
Row-sparse MMV [4]	11.8	14.3	15.3
Proposed row-sparse rank-deficient MMV	13.7	15.6	16.4

**Table 2.** *In vivo*: SNR for same Fourier mapping with all echoes.

**Recovery Method**	**32 Lines**	**48 Lines**	**64 Lines**
Row-sparse MMV [4]	8.8	14.2	17.9
Proposed row-sparse rank-deficient MMV	10.7	15.7	19.1

**Table 3.** *Ex vivo*: SNR for different Fourier mappings with different echoes.

**Recovery Method**	**32 Lines**	**48 Lines**	**64 Lines**
Group-sparse [4]	12.7	15.2	16.7
Proposed group-sparse rank-deficient	14.6	16.7	18.1

**Table 4.** *In vivo*: SNR for different Fourier mapping with different echoes.

**Recovery Method**	**32 Lines**	**48 Lines**	**64 Lines**
Group-sparse [4]	10.7	16.3	20.3
Proposed group-sparse rank-deficient	12.6	17.7	21.5

## Appendix

Here we derive the algorithm for solving:

(A1) $$\underset{x}{\text{min}}{\left\|x\right\|}_{2,1}+\gamma {\left\|X\right\|}_{NN}s.t.{\left\|y-Ax\right\|}_{2}^{2}\leq ɛ$$

where x, X, y, and A in Equation (A1) stand for α, A, vec(Y) and FΦ in Equation (10) respectively.

Solving the constrained optimization problem directly is difficult. Therefore we propose to solve its unconstrained Lagrangian form:

(A2) $$\underset{x}{\text{min}}{\left\|y-Ax\right\|}_{2}^{2}+\lambda {\left\|x\right\|}_{2,1}+\lambda \gamma {\left\|X\right\|}_{NN}$$

where λ is the Lagrangian multiplier.

There exists a relationship between the constrained form Equation (A1) and the unconstrained version Equation (A2) for proper choice of λ given ε. Unfortunately the relationship is not analytical. Thus in general, it is not possible to predict λ given the value of ε. It must be obtained iteratively. At a later stage we will show how the constrained problem Equation (A2) is progressively cooled (by reducing λ) to reach the solution of the original constrained problem Equation (A1). But for now, we concentrate on the solution of Equation (A2).

First, we replace the Nuclear Norm in Equation (A2) by its equivalent Ky-Fan norm, *i.e.*, ‖*X*‖*_NN_* =*Tr* (*X^H^X*) ^1/2^, Thus, we will solve the following equivalent problem:

(A3) $$\underset{x}{\text{min}}{\left\|y-Ax\right\|}_{2}^{2}+\lambda {\left\|x\right\|}_{2,1}+\lambda \gamma Tr{\left(X^{H}X\right)}^{1/2}$$

We follow the Majorization Minimization (MM) approach to solve this problem [17]. The generic MM algorithm is as follows:

Let *J(x)* be the (scalar) function to be minimized:

(1) Set iteration count *k* = *0* and initialize *x_0_*.
  Repeat Steps 2–4 until a suitable stopping criterion is met.
(2) Choose *G_k_(x)* such that
  (a) *G_k_* (*x*) ≥ *J* (*x*) for all *x*.
  (b) *G_k_* (*x*) = *J* (*x*).
(3) Set *x_k_*_+_*_1_* as the minimizer for *G_k_(x)*.
(4) Set k = k + 1, go to Step 2.

For our problem the function to be minimized is: *J(x)* = ‖*y* − *Ax*‖_2_ + λ ‖*x*‖_2,1_ + *λγTr* (*X^H^X*)^1/2^. There is no closed form solution to *J(x)*, it must be solved iteratively. At each iteration we choose:

(A4) $$G_{k}(x)={\left\|y-Ax\right\|}_{2}^{2}+{\left(x-x_{k}\right)}^{H}\left(aI-A^{H}A\right)(x-x_{k})+\lambda {\left\|x\right\|}_{2,1}+\lambda \gamma T_{r}{\left(X^{H}X\right)}^{1/2}$$

*G_k_(x)* satisfies the condition for MM algorithm when *a* > max*eigenvalue* (*A^H^A*).

Now *G_k_(x)* can be alternately expressed as follows:

(A5) $$G_{k}(x)=a{\left\|x_{k}+\frac{1}{a}A^{H}(y-Ax_{k})-x\right\|}_{2}^{2}+\lambda {\left\|x\right\|}_{2,1}+\lambda \gamma T_{r}{\left(X^{H}X\right)}^{1/2}$$

where *K* consists of terms independent of *x*.

Minimizing Equation (A5) is the same as minimizing the following:

(A6) $$G_{k}^{\prime }(x)={\left\|b-x\right\|}_{2}^{2}+\frac{\lambda }{a}{\left\|x\right\|}_{2,1}+\lambda \gamma T_{r}{\left(X^{H}X\right)}^{1/2}$$

Where $b=x_{k}+\frac{1}{a}A^{H}(y-Ax_{k})$.

The problem now is to minimize Equation (A6). To do so, we take the derivative of the function:

(A7) $$\nabla G_{k}^{\prime }(x)=2x-2b+\frac{\lambda }{a}Qx+\frac{\lambda \gamma }{2a}{\left(X^{H}X\right)}^{-1/2}X$$

where *Q* = *Diag*(1./‖*x*‖_2_); “.” denotes element wise division.

Setting the gradient to zero, one gets:

(A8) (I+D)x=b

Where $Q=\frac{\lambda }{2a}\text{Diag}(1./{\left\|x\right\|}_{2})+\frac{\lambda \gamma }{4a}I⊗{\left(X^{H}X\right)}^{-1/2}$.

The problem Equation (A8) represents a system of linear equations. It should be noted that the system (*I* + *D*) is symmetric. Hence it can be efficiently solved by newly developed MINRES-QLP algorithm [18].

Based on this derivation, we propose the following algorithm to solve (12 or 13):
Intitialize: *x*_0_ = 0 Repeat until: ${\left\|y-Ax\right\|}_{2}^{2}\geq ɛ$ Step 1. $b=x_{k}+\frac{1}{a}A^{H}(y-Ax_{k})$ Step 2. $D=\frac{\lambda }{2a}\text{Diag}(1./{\left\|x\right\|}_{2})+\frac{\lambda \gamma }{4a}I⊗{\left(X^{H}X\right)}^{-1/2}$ Step 3. Compute *x* by solving (*I* + *D*)*x* = *b* End

Our discussion so far had been on solving the unconstrained problem Equation (A2). We propose to solve the constrained problem Equation (A1) by iteratively solving a sequence of unconstrained problems Equation (A2) by reducing the value of λ. This technique is called ‘cooling’ and has been used previously to solve similar problems in constrained optimization [19,20]. The cooling technique solves the constrained problem in two loops. The outer loop decreases the value of λ. The inner loop solves the unconstrained problem for a specific value λ. As the value of λ is progressively decreased, the solution of the unconstrained problem reaches the desired solution. Such a cooling technique works because the pareto curve between the objective function and the constraint is smooth [21]:
Intitialize: *x*_0_ = 0, *λ* = max(*A^T^ y*) Choose a decrease factor for cooling λ. Outer Loop: While ${\left\|y-Ax\right\|}_{2}^{2}\geq ɛ$ Inner Loop: While ^2^ $\frac{J(x_{k-1})-J(x_{k})}{J(x_{k+1})+J(x_{k})}\geq \mathrm{Tol}$ Step 1. $b=x_{k}+\frac{1}{a}A^{H}(y-Ax_{k})$ Step 2. $D=\frac{\lambda }{2a}\text{Diag}(1./{\left\|x\right\|}_{2})+\frac{\lambda \gamma }{4a}I⊗{\left(X^{H}X\right)}^{-1/2}$ Step 3. Compute *x* by solving (*I* + *D*)*x* = *b* End While^2^ *λ* = *λ*×*DecreaseFactor* End While^1^

The stopping criterion here is based on the data consistency term. The iterations stop when $∥y-Ax{∥}_{2}^{2}\leq ɛ$. Here *ε* is defined as the product of the number of pixels in each echo times the number of echoes times the variance of noise (*η*). The decrease factor is fixed at 0.5. The tolerance (Tol) is fixed at 10^−3^. The maximum number of iterations that are allowed for the inner and the outer loops are 50 and 10 respectively.
